# Influence of Curing Mode on the Surface Energy and Sorption/Solubility of Dental Self-Adhesive Resin Cements

**DOI:** 10.3390/ma10020129

**Published:** 2017-02-04

**Authors:** Hyun-Jin Kim, Rafat Bagheri, Young Kyung Kim, Jun Sik Son, Tae-Yub Kwon

**Affiliations:** 1 Department of Dental Science, Graduate School, Kyungpook National University, Daegu 41940, Korea; khjin0815@knu.ac.kr; 2 Dental Materials Department and Biomaterials Research Centre, Shiraz University of Medical Sciences, Shiraz 7183643111, Iran; 3 Department of Conservative Dentistry, School of Dentistry, Kyungpook National University, Daegu 41940, Korea; wisekim@knu.ac.kr; 4 Korea Textile Development Institute, Daegu 41842, Korea; sonjk1@empas.com; 5 Department of Dental Biomaterials, School of Dentistry, Kyungpook National University, Daegu 41940, Korea

**Keywords:** polymerization, self-adhesive resin cement, solubility, sorption, surface energy

## Abstract

This study investigated the influence of curing mode (dual- or self-cure) on the surface energy and sorption/solubility of four self-adhesive resin cements (SARCs) and one conventional resin cement. The degree of conversion (DC) and surface energy parameters including degree of hydrophilicity (DH) were determined using Fourier transform infrared spectroscopy and contact angle measurements, respectively (*n* = 5). Sorption and solubility were assessed by mass gain or loss after storage in distilled water or lactic acid for 60 days (*n* = 5). A linear regression model was used to correlate between the results (%DC vs. DH and %DC/DH vs. sorption/solubility). For all materials, the dual-curing consistently produced significantly higher %DC values than the self-curing (*p* < 0.05). Significant negative linear regressions were established between the %DC and DH in both curing modes (*p* < 0.05). Overall, the SARCs showed higher sorption/solubility values, in particular when immersed in lactic acid, than the conventional resin cement. Linear regression revealed that %DC and DH were negatively and positively correlated with the sorption/solubility values, respectively. Dual-curing of SARCs seems to lower the sorption and/or solubility in comparison with self-curing by increased %DC and occasionally decreased hydrophilicity.

## 1. Introduction

Self-adhesive resin cements (SARCs) have been developed to reduce the multiple clinical steps required for application of the luting agents. These materials include acidic, hydrophilic methacrylate monomers, which simultaneously demineralize and infiltrate the tooth substrate, resulting in micromechanical retention and potentially additional chemical bonding [1,2]. The use of SARCs also reduces the occurrence of post-operative sensitivity [2].

In general, the SARCs are categorized as dual-cured resin cements, in which both chemical-activating and light-activating mechanisms are involved. The degree of conversion (DC) of dual-cured resin cements is generally lower when they are self-cured than when dual-cured, although the extent is related to the initiation system in each material [3]. In particular, acidic monomers incorporated in the SARCs may lower the DC even in their dual-cure mode because they may chemically interact with the amine initiator in the materials [3]. Thus, some commercial SARCs include proprietary activator/initiator systems to overcome this incompatibility [3,4]. Nonetheless, the acidic nature of an SARC has been raised as one factor hampering its effective polymerization, especially under chemical polymerization [5].

In the oral environment, the sensitivity of resin cement to moisture increases, raising the possibility of deboning and dissolution of the material at the marginal gap, consequently resulting in weakening and fracture of the indirect restoration [1]. An increased sorption/solubility may present early hydrolytic degradation, which reduces the lifetime of indirect restorations [6]. Conventional resin cements are based on crosslinking monomers such as bisphenol A diglycidyl methacrylate (Bis-GMA), triethylene glycol dimethacrylate (TEGDMA), and urethane dimethacrylate (UDMA) are basically hydrophobic [7]. SARCs are more hydrophilic than conventional resin cements due to the incorporation of acidic monomers in specific concentrations [1,7], suggesting that such resin cements are more susceptible to water sorption and solubility [8].

Commercial SARCs, which vary greatly in their composition, have a wide range of surface energy characteristics including surface hydrophilicity/hydrophobicity [7], thereby resulting in different sorption/solubility one another [8]. In addition, the curing protocol of SARCs may affect the surface energy, when considering possible differences in the polymer network and residual monomers [9], thus also potentially affecting the degradation of the materials. However, there are few studies regarding the influence of SARC curing methods on the surface energy characteristics and, as a result, sorption/solubility.

Therefore, the purpose of this in vitro study was to investigate the influence of two curing modes (either dual- or self-cure) on the surface energy and sorption/solubility of four SARCs (RelyX U200, RXU; Maxcem Elite, MCE; BisCem, BC; and Multilink Speed, MLS) and one conventional resin cement (Duo-Link, DL) (as the control). Their codes, brand names, manufacturers, and main compositions are shown in Table 1. The DCs and surface energy parameters of each resin cement polymerized in either dual- or self-cure mode were calculated. The sorption and solubility of five resin cements were investigated after immersing them in either distilled water or lactic acid for 60 days. The null hypotheses tested were that the different curing modes do not affect (1) the surface energy parameters nor (2) the sorption/solubility values of the materials tested.

## 2. Results

### 2.1. Degree of Conversions (DC)

**Figure 1:**
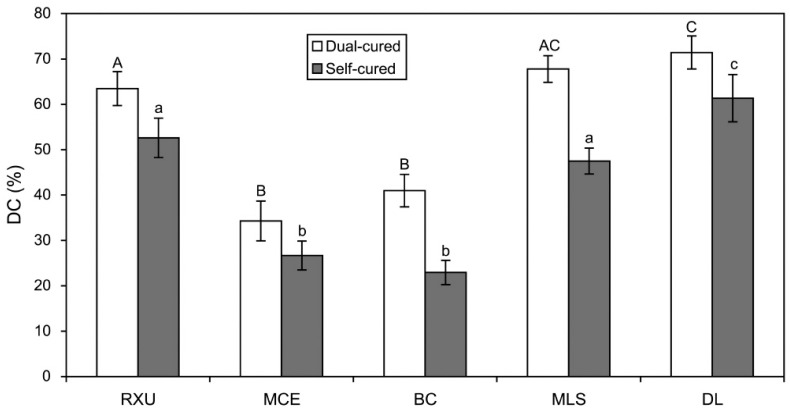
Degree of conversion (DC) of the resin cements polymerized in either dual- or self-cure mode (*n* = 5). The vertical bars indicate standard deviations. Upper- and lower-case letters indicate statistically similar means within the dual-cured and self-cured specimens, respectively (*p* > 0.05). For all materials, there were significant differences in the value between the two curing modes (*p* < 0.05).

Figure 1 represents the %DC of the five resin cements, which underwent dual-curing with light irradiation or self-curing without light irradiation. For all the resin cements tested, the dual-cure mode consistently produced significantly higher %DC values than the self-cure mode (*p* < 0.05). The conventional resin cements DL exhibited the highest %DC values in both curing modes (*p* < 0.05). BC and MCE showed significantly lower %DC values than the other materials, regardless of the curing mode applied (*p* < 0.05), with no statistical differences from each other (*p* > 0.05).

### 2.2. Surface Energy Parameters

**Figure 2:**
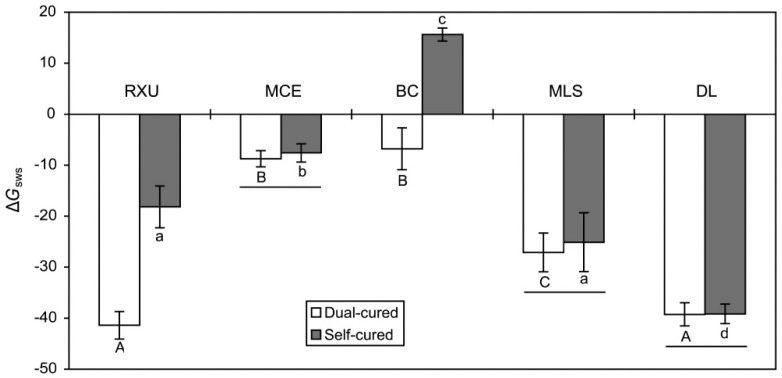
Degree of hydrophilicity (expressed as Δ*G*_sws_) of the resin cements tested (*n* = 5). The same upper- and lower-case letters indicate statistically similar means within the dual- and self-cured specimens, respectively (*p* > 0.05). The horizontal bars connect statistically similar means between the two curing modes within each material (*p* > 0.05).

When the surface roughness of the resin cements was checked prior to the CA measurements, the mean *R*_a_ values ranged from 0.03 to 0.09 μm (data not shown). The surface energy parameters of the materials tested are shown in Table 2 and Figure 2. For DL, there was no significant difference in each surface energy component between the two curing modes (*p* > 0.05). For the SARCs, on the other hand, significant differences in each component between the curing modes were found (*p* < 0.05), with the exception of *γ*_s_*^−^* values in MCE and MLS (*p* > 0.05). All the resin cements tested consistently showed large *γ*_s_^LW^ and small *γ*_s_^AB^ values, *γ*_s_^−^ values being consistently greater than *γ*_s_*^+^* values. As shown in Figure 2, all the resin cements showed hydrophobic characteristics (negative Δ*G*_sws_), with the exception of self-cured BC (positive Δ*G*_sws_). MCE, MLS, and DL did not show significant differences in the value between the two curing modes (*p* > 0.05), whereas self-cured RXU and BC were significantly more hydrophilic than dual-cured ones (*p* = 0.008).

**Figure 3:**
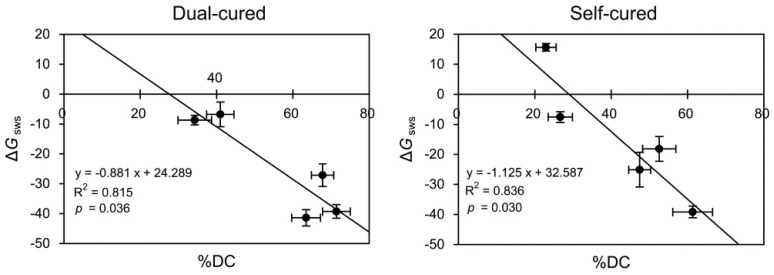
Graphs of linear regression of degree of hydrophilicity (Δ*G*_sws_) as a function of degree of conversion (%DC). The horizontal and vertical bars indicate standard deviations.

Figure 3 shows the graphs of linear regression of Δ*G*_sws_ as a function of %DC in the two curing modes. For both modes, significant negative linear regressions were established between the two parameters (dual-cured: R^2^ = 0.815, *p* = 0.036; self-cured: R^2^ = 0.836; *p* = 0.030).

### 2.3. Sorption and Solubility

**Figure 4:**
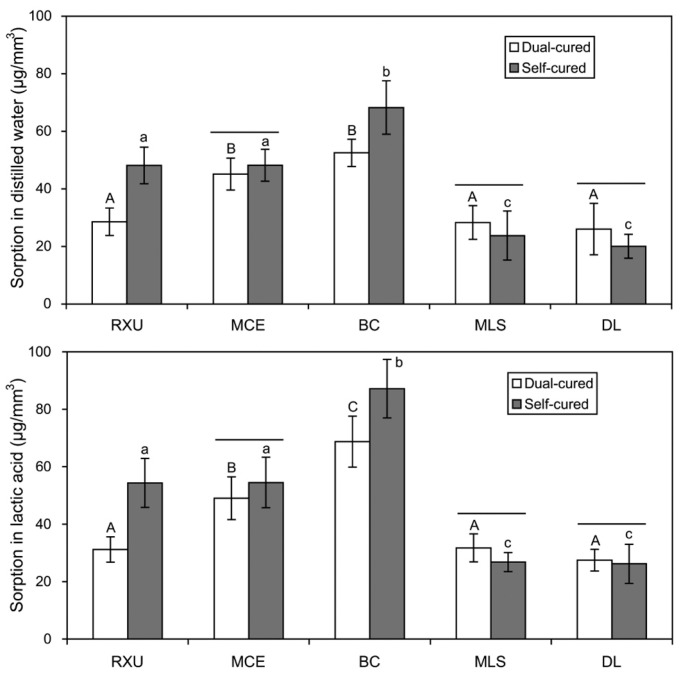
Sorption (μg/mm^3^) of the resin cements polymerized in either dual- or self-cure mode (*n* = 5). The vertical bars indicate standard deviations. The same upper- and lower-case letters indicate statistically similar means within the dual- and self-cured specimens, respectively (*p* > 0.05). The horizontal bars connect statistically similar means between the two curing modes within each material (*p* > 0.05).

**Figure 5:**
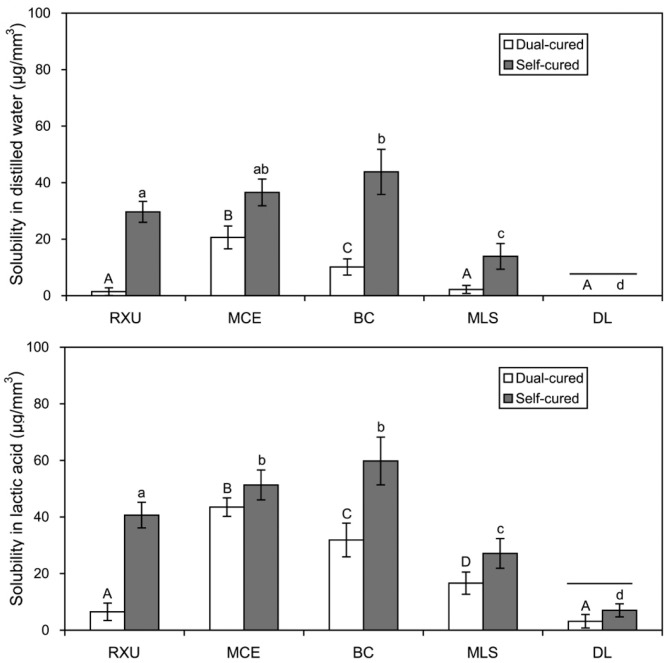
Solubility (μg/mm^3^) of the five resin cements polymerized in either dual- or self-cure mode (*n* = 5). The vertical bars indicate standard deviations. The same upper- and lower-case letters indicate statistically similar means within the dual- and self-cured specimens, respectively (*p* > 0.05). The horizontal bars connect statistically similar means between the two curing modes within each material (*p* > 0.05).

The sorption and solubility values of the resin cements immersed in either distilled water or lactic acid for 60 days are presented in Figure 4 and Figure 5. Overall, the conventional resin cement DL exhibited the lowest sorption/solubility, with no statistical differences between the two curing modes (*p* > 0.05). On the contrary, the self-cured BC exhibited the highest sorption and solubility values. As for MCE and MLS, there were no significant differences in the sorption between the two curing modes (*p* > 0.05, Figure 4), whereas the self-cure mode produced significantly higher solubility than the dual-cure mode (*p* < 0.05, Figure 5).

### 2.4. Linear Regressions

**Figure 6:**
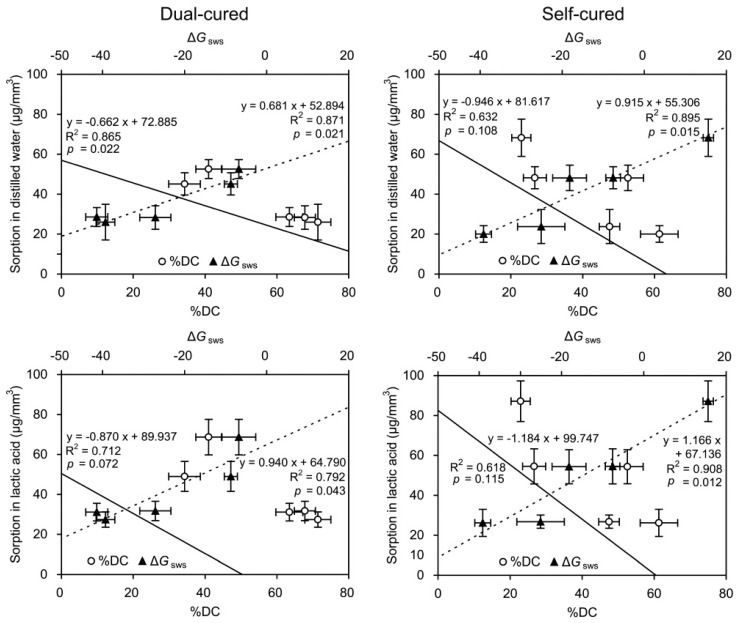
Graphs of linear regression of sorption (μg/mm^3^) as a function of degree of conversion (%DC) and degree of hydrophilicity (Δ*G*_sws_). The horizontal and vertical bars indicate standard deviations.

**Figure 7:**
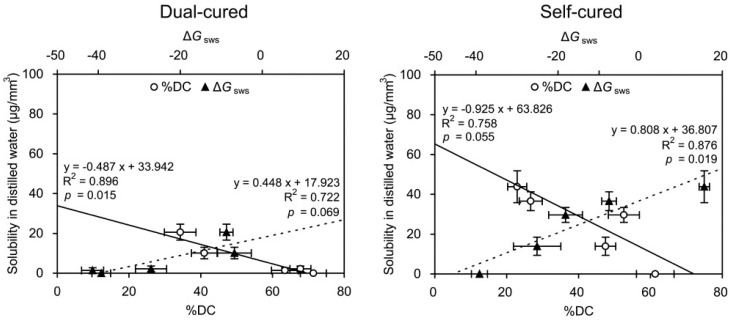

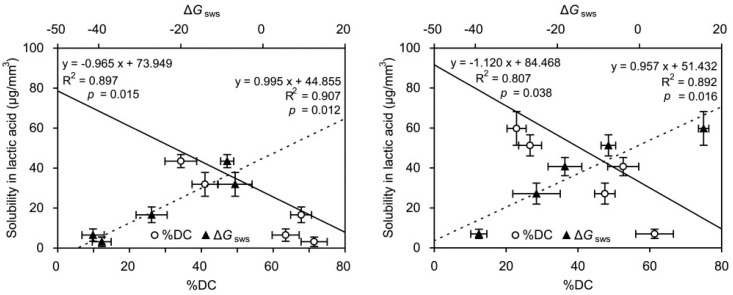
Graphs of linear regression of solubility (μg/mm^3^) as a function of degree of conversion (%DC) and degree of hydrophilicity (Δ*G*_sws_). The horizontal and vertical bars indicate standard deviations.

Figure 6 and Figure 7 show the graphs of linear regression of sorption/solubility as a function of %DC/Δ*G*_sws_. For all cases, negative (%DC) or positive (Δ*G*_sws_) linear regressions were established between the %DC/Δ*G*_sws_ and the sorption/solubility values. The Δ*G*_sws_ values consistently produced higher R^2^ values than the %DC values for the sorption (Figure 6), whereas this tendency was less distinct for the solubility (Figure 7).

## 3. Discussion

In the present study, four SARCs, containing various phosphoric ester monomers, and one conventional resin cement, were tested (Table 1). The hydrophobic crosslinking monomers Bis-GMA, TEGDMA, and UDMA in the resin cements also have polar hydrophilic sites (hydroxyl, ethylene oxide, and urethane groups, respectively), which can serve as water absorption sites [10,11], leading to swelling of the cured resin as well as increased hydrolysis susceptibility [12]. Moreover, the functional groups (e.g., phosphate and carboxyl groups) in acidic monomers, which are commonly incorporated into self-etching resin cements and SARCs, usually exhibit strong hydrophilic characteristics [12], potentially resulting in high sorption and solubility after polymerization [1].

The surface roughness of the resin cements was checked prior to measuring the CAs because changes in *R*_a_ above 0.1 μm can influence the value, thereby altering the surface energy characteristics [13,14,15]. In this study, there were only small variations in *R*_a_ (below 0.1 μm) among the resin cement surfaces prepared against a Mylar polyester film.

In general, the surface energy parameters of resin-based materials are calculated from the CA values measured on polymerized surfaces because it is actually impossible to make an unpolymerized surface flat and smooth [7,11,14,16]. In this case, the basic assumption is that the surface energy parameters of uncured and cured resin materials would be similar since all groups and segments of the functional monomers are also present in the polymer, with the exception of the vinyl groups [14,16]. However, the findings of this study suggest the surface hydrophilicity of some SARCs (RXU and BC in this study) are significantly altered depending on the curing mode used (Figure 2). The conventional resin cement (DL) did not exhibit a significant difference in Δ*G*_sws_ between the two curing modes, notwithstanding a significant difference in %DC (Figure 1). RXU and BC exhibited significantly lower Δ*G*_sws_ (more hydrophobic) in the dual-cure mode than in self-cure mode, likely due to significantly lowered strength of *γ*_s_*^−^* in the dual-cure mode (Table 2). On the contrary, MCE and MLS did not demonstrate significant differences in Δ*G*_sws_ between the two curing modes. These findings imply that, in some cured SARCs, different arrangements of functional groups of the acidic monomers during polymerization (depending on the curing mode used) may result in differences in the surface energy characteristics between the two curing modes [9]. Since the influence of curing mode on surface energy parameters was found to be material-dependent, the first hypothesis has to be partially rejected.

Taking Δ*G*_sws_ = 0 as the boundary between hydrophobicity and hydrophilicity [17], all the dual- or self-cured resin cements tested were found to present a mainly hydrophobic character with the exception of the self-cured BC (Figure 2). As seen in Figure 3, the DHs (expressed as Δ*G*_sws_) of the resin cements were strongly negatively correlated with the %DCs in both curing modes. The high R^2^ (coefficient of determination) values suggest that Δ*G*_sws_ may serve as a surrogate measure of DC of the resin cement. However, further research is needed to determine how surface energy changes as polymerization progresses [18].

When resin-based composites are immersed in aqueous solutions, a solvent such as water can intermingle with the polymer matrix physically (plasticization) and chemically (hydrolysis and degradation) [1,19]. The solvent diffuses into the resin network and reduces the intermolecular interaction of the polymer chains (swelling), yielding an increase in weight (sorption) [1]. Meanwhile, solubility in a resin-based composite reflects the amount of released residual or leachable monomers as well as filler particles from its surface, resulting in loss of weight [1,20]. The release of uncured monomers also presents a potentially sensitizing and irritating factor for the oral tissues [21].

As seen in Figure 4 and Figure 5, the sorption and solubility values of the resin cements polymerized in either dual- or self-cure mode were generally affected by the DC (Figure 1) and/or surface energy parameters (Table 2 and Figure 2). Therefore, the second hypothesis that the curing modes do not affect the sorption/solubility of the resin cements was rejected. In the Lewis acid–base interactions, the Lewis acid (*γ*_s_^+^) and the Lewis base (*γ*_s_*^−^*) components can be regarded as hydrogen bond donating and accepting, respectively [7,14]. All luting cements tested showed large *γ*_s_*^−^* values and very small *γ*_s_^+^ values (Table 2), indicating a predominantly hydrogen bond accepting character rather than donating character [14]. Water, as a solvent, can establish hydrogen bonds with the polar sites (in particular, hydrogen accepting sites) of polymer networks or even residual unreacted monomers, which are present in the polymer networks or trapped in voids and pores during polymerization, in the cured resin-based composite [1]. Furthermore, resin-based composites containing higher concentrations of acidic monomers having higher polarity can develop a higher level of hydrogen bonds with water, thereby increasing the liquid uptake [1,14].

Distilled water and lactic acid were chosen as storage media in this study. Artificial saliva may simulate the liquids that constantly interact with teeth and restorations in the mouth in a more clinically relevant manner. It has been shown that water and artificial saliva are generally comparable as storage media in terms of water sorption [22]. However, the continued adsorption of artificial saliva components may mask the actual solubility of the resin materials [22]. Lactic acid is one of the main acids produced by human dental plaque [20], which may accelerate surface degradation of resin-based composites [23,24]. In this study, the effect of lactic acid on the resin cements was generally more deleterious than that of distilled water in the degradation of the materials (Figure 4 and Figure 5), in accordance with the results of previous studies [1,20,25,26]. A study by da Silva et al. [20] reported that the highest diffusion coefficient occurred when resin-based composites were immersed in lactic acid, indicating a faster diffusion of lactic acid inside the resins than distilled water. Lactic acid with the polar functional groups (hydroxyl (–OH) and carboxyl (–COOH) groups) may form more hydrogen bonds with resin cements, particularly with SARCs, as compared to water [1]. Such hydrogen bonding potential of the phosphoric ester monomers may also depend on their chemical structures [14].

In this study, BC exhibited the greatest sorption and solubility values among the four SARCs tested (Figure 4 and Figure 5), which can partly be attributed to the presence of HEMA, together with acidic monomers, in its composition (Table 1). HEMA is a highly hydrophilic monomer used for enhanced adhesion promotion [12,14]. Nevertheless, HEMA will readily absorb water both in uncured and even cured states [12]. The conventional resin cement DL, not having any acidic monomers, showed only a low degree of sorption/solubility. Slight water sorption sometimes may be favorable in compensating resin polymerization shrinkage, thus relieving internal shrinkage stresses and potentially improving marginal seal [1].

The regression analysis results (Figure 6 and Figure 7) clearly show that the slope of the prediction line of the sorption/solubility decreased and increased linearly with increasing %DC and Δ*G*_sws_, respectively. In general, the DC of a resin-based composite provides valid information about the durability and biological safety of the material because it affects the mechanical properties and degradation by water and/or oral acids [21,27]. As seen in Figure 6, the consistently steeper slope of the lines (higher R^2^ values) suggests that the sorption characteristics of the resin cements could be better explained by the variations in the Δ*G*_sws_ than by those in the %DC among the materials. In regard to solubility, such predominant effects of Δ*G*_sws_ values were less distinct (Figure 7). MCE and MLS showed statistically similar sorption values in both immersion media (Figure 4), but significantly higher solubility values were found in the self-cure mode than in the dual-cured mode (Figure 5). The solubility behavior of a resin cement may instead be related to the elution of unreacted components, the amount of which is inversely proportional to the DC [1]. The findings of this study suggest that surface energy characteristics should be taken into account in the analysis of resin material degradation. Measuring CA might be regarded as a simple method for roughly estimating the sorption/solubility behavior of resin cements, together with DC.

Kim et al. [14] demonstrated that the shear bond strength of various luting cements to zirconia ceramic was positively correlated with the base components (*γ*_s_*^−^* values) of the materials. The base components of a resin-based composite seem to govern the DH (Table 2 and Figure 2) [11]. In this study, MCE, MLS, and DL, with no significant differences in the *γ*_s_*^−^* value between the two curing modes, also showed no significant differences in Δ*G*_sws_ value. Kim et al. [11] reported that the more hydrophobic SARCs produced higher shear bond strengths to dentin than the more hydrophilic ones, indicating that large base components (high *γ*_s_*^−^* values) and, as a result, increased DH, do not necessarily indicate enhanced bonding to a dental substrate. Such high DH of SARCs also is unfavorable in terms of sorption/solubility. Therefore, SARCs with optimal surface energy characteristics should be formulated to enhance the bonding behavior but decrease the sorption/solubility.

The findings of this study suggest that commercial SARCs may suffer a higher degradation than conventional resin cements in the oral environment, especially when the materials are partially polymerized by self-curing [28]. Therefore, dual-cured resin cements—including SARCs—should achieve their maximum DC and, if possible, high surface hydrophobicity to withstand such intraoral challenges [29]. Increased water sorption of a resin cement can degrade its mechanical properties by the plasticizing effect and also by dissolving and leaching out of unreacted monomers and filler particles [9,19,29,30]. Changes in bulk properties of the resin-based composite after immersion in a solution were not included in this study; further investigation is needed.

## 4. Materials and Methods

### 4.1. Degree of Conversions (DC)

The %DC of the resin cements was determined using a Fourier transform infrared (FTIR) spectroscope (IRPrestige-21, Shimadzu Corp, Kyoto, Japan). A total of 50 (*n* = 5 per group) disc-shaped specimens (6 mm in diameter and 1 mm in thickness) were prepared by polymerizing them in either dual- or self-cure mode, as follows. Cylindrical molds were placed on a Mylar polyester film over a glass slide (dual-cure) or metal plate (self-cure). Each resin cement was mixed in accordance with the corresponding instructions, filled into the mold, and then covered with another Mylar polyester film and then glass slide (dual-cure) or metal plate (self-cure) to form an assembly. In dual-cured groups, the materials were light-irradiated for 40 s by placing the light guide tip of a curing light (Bluephase^®^ 20i, Ivoclar Vivadent, Schaan, Liechtenstein) against the upper glass slide. The second side of the resin specimen was irradiated in the same way as the first side [31]. During the experiment, the output intensity (2000 mW/cm^2^) was constantly monitored by a radiometer. The assembly was then transferred to a 37 °C oven. The specimen was removed from the mold 15 min after the start of irradiation [31], and the periphery was finished using silicon carbide paper to remove flash and irregularities. In the self-cured groups, the assembly was clamped together and transferred to the oven. The specimen was removed from the mold 60 min from the start of mixing [31], and the periphery was finished as described above. The dual- or self-cured specimens were stored in the oven until the start of testing (24 h after the start of irradiation (dual-cured) or mixing (self-cured)).

Each specimen was pressed against an attenuated total reflectance (ATR) prism (MIRacle, Pike Technologies Inc., Madison, WI, USA) in the FTIR instrument, and the absorbance spectrum was acquired by scanning 20 times over a 4000–700 cm^−1^ range with a resolution of 4 cm^−1^. Uncured material from each resin cement was also scanned, its spectrum being used as the uncured reference. The aliphatic C=C peak at 1638 cm^−1^ was acquired, while either the aromatic C=C peak at 1608 cm^−1^ (RXU, DL) or the N–H peak at 1537 cm^−1^ (MCE, BC, and MLS) were used as the internal calibration [32]. The %DC was determined by comparing the height of the peaks for the methacrylate vinyl group (aliphatic C=C) in the cured material against that in the uncured material, using the following equation: DC (%) = (1 − *A*_c_/*A*_uc_) × 100, where *A*_c_ and *A*_uc_ are the peaks for the cured and uncured materials, respectively.

### 4.2. Surface Energy Parameters

To investigate the surface energy of the resin cements, contact angles (CAs) of a liquid triplet were measured on the surfaces of the materials polymerized in either dual- or self-cure mode. CAs were determined on cured resin specimens, which had an outermost surface free of filler when cured in contact with a polyester film [16].

Prior to measuring the CAs, the surface roughness of the resin cements was checked because changes in roughness can alter the CA [13,14]. Five cured specimens for each group were prepared as described in Section 4.1. Average surface roughness (*R*_a_) was measured using a profilometer (Surftest SV-400, Mitutoyo Corp., Kawasaki, Japan). The stylus speed, cutoff, and range used were 0.1 mm/s, 0.25 mm, and 600 μm, respectively. Three measurements were performed on each resin specimen, and the average value was used.

For the CA measurements, five disc-shaped cured specimens for each group were prepared as described in Section 4.1. Water (W: *γ*: 72.8; *γ*^LW^: 21.8; *γ^+^*: 25.5; *γ*^−^: 25.5), glycerol (G: *γ*: 64; *γ*^LW^: 34; *γ^+^*: 3.92; *γ*^−^: 57.4), and methylene iodide (MI: *γ*: 50.8; *γ*^LW^: 50.8) were used as the test liquids (all in mJ·m^−2^) [7,33]. The CA of each of the three liquids was measured on the surfaces by the sessile drop method using a CA goniometer (OCA 15 plus, DataPhysics Instrument GmbH, Filderstadt, Germany). The CA measurements were carried out at 23 ± 1 °C with relative humidity at 50% ± 5% [34].

Surface energy components of a solid were calculated using the Young-Dupré equation combined with the Lifshitz-van der Waals/Lewis acid-base theory [7,14]: *γ*_l_(1 + cos*Θ*) = 2[(*γ*_s_^LW^*γ*_l_^LW^)^1/2^ + (*γ*_s_^+^*γ*_l_^−^)^1/2^ + (*γ*_s_*^−^γ*_l_^+^)^1/2^], where *Θ* is the contact angle; *γ*_l_ and *γ*_s_ are the surface tensions of the liquid (l) and solid (s) surfaces, respectively; and the superscripts LW, +, and − refer to the Lifshitz-van der Waals, acid, and base components, respectively. The total surface energy *γ*_s_ was derived by the equation [7,14]: *γ*_s_ = *γ*_s_^LW^ + *γ*_s_^AB^ = *γ*_s_^LW^ + 2(*γ*_s_^+^*γ*_s_^−^)^1/2^, where *γ*_s_^AB^ is the acid/base component.

The degree of hydrophilicity (DH) of the materials was further investigated using thermodynamic notation. The work of cohesion, *W*_c_, is expressed by using the free energy, *G*, so that Δ*G*_c_ = −2*γ* = −*W*_c_ [35]. The DH of a material is related to the magnitude of Δ*G*_sws_ = −2*γ*_sw_, where *γ*_sw_ = *γ*_sw_^LW^ + *γ*_sw_^AB^, in which w indicates water. The *γ*_sw_^LW^ and the *γ*_sw_^AB^ were calculated using the equations, respectively [17]: *γ*_sw_^LW^ = [(*γ*_s_^LW^)^1/2^ − (*γ*_w_^LW^)^1/2^]^2^ and *γ*_sw_^AB^ = 2[(*γ*_s_^+^*γ*_s_*^−^*)^1/2^ + (*γ*_w_^+^*γ*_w_^−^)^1/2^ − (*γ*_s_^+^*γ*_w_^−^)^1/2^ − (*γ*_w_^+^*γ*_s_^−^)^1/2^]. Δ*G*_sws_ = 0 was considered as the boundary between hydrophobicity and hydrophilicity [17]. When Δ*G*_sws_ is positive, the surface of the material is hydrophilic (high DH); when Δ*G*_sws_ is negative, that of the material is hydrophobic (low DH) [17].

### 4.3. Sorption and Solubility

For the sorption/solubility test, 10 specimens were prepared for each group (five for immersion in distilled water and five in lactic acid) as described in Section 4.1 [25]. The specimens were stored in two desiccators (at 37 °C for 22 h and then at 23 °C for 2 h) and repeatedly weighed after 24-h intervals using a balance (DVG214C, Ohaus Corp., Parsippany, NJ, USA) to an accuracy of 0.1 mg until a constant mass (*m*_1_) was obtained [31]. After calculating the volume (*V*, in mm^3^) of the specimens by measuring the dimensions using a digital caliper, the specimens were individually placed in lightproof glass vials containing either distilled water (pH = 7) or 0.01 M lactic acid (pH = 4) maintained at 37 °C [1,20]. Fresh distilled water and lactic acid solutions were replaced daily to avoid variation in pH [1]. After 60 days, the specimens were removed from the immersion solutions and gently rinsed with water. Visible surface liquid was then removed by blotting with absorbent paper and waving the specimens in the air [31], and *m*_2_ was recorded. The specimens were then placed in the desiccators again, and reweighed until a constant mass (*m*_3_) was obtained. Sorption (*S*_P_) and solubility (*S*_L_) during the 60-day immersion, in ug/mm^3^, were calculated using the following formulae [1,20]: *S*_P_ = (*m*_2_ − *m*_3_)/*V*; *S*_L_ = (*m*_1_ − *m*_3_)/*V*.

### 4.4. Statistical Analysis

For all data which did not satisfy the equal variance assumption (Leven’s test), non-parametric statistical procedures were applied. The Kruskal-Wallis test was employed among resin cements within each curing mode, followed by the Mann-Whitney post hoc test, with adjustment of significance levels using the Benjamini and Hochberg method of a false discovery rate; between two curing modes within each resin cement, the Mann-Whitney test was used [36]. Linear regression analyses were performed to correlate (1) the %DC with the DH and (2) the %DC/DH with the sorption/solubility for each curing mode. The statistical analysis was performed using SPSS 17.0 for Windows (SPSS Inc., Chicago, IL, USA) at *α* = 0.05.

## 5. Conclusions

This in vitro study investigated the influence of two curing modes on the surface energy and sorption/solubility of four SARCs (RXU, MCE, BC, and MLS) and one conventional resin cement (DL) (as the control). Within the limitations of this study, the following findings were noted:

- For all the resin cements tested, the dual-cure mode consistently produced significantly higher %DC values than the self-cure mode.
- Dual-curing of the two SARCs (RXU and BC) resulted in greater surface hydrophobicity as compared with self-curing, whereas curing mode had no significant effect in the other two SARCS (MCE and MLS) or in the conventional resin cement (DL).
- Overall, the SARCs exhibited greater sorption/solubility values than the conventional resin cement (DL), especially when self-cured. The effect of lactic acid on the materials was generally more deleterious than that of distilled water.

## Figures and Tables

**Table 1:** Materials used in the present study.

Code	Brand Name	Manufacturer	Composition * (Batch Number)	Filler Loading/Average Particle Size *
RXU	RelyX U200	3M ESPE, Seefeld, Germany	Base: Mono-, di-, and tri-glycerol esters of phosphoric acid dimethacrylate, TEGDMA, glass, silica, sodium persulfate, tert-butyl peroxy-3,5,5-trimethylhexanoate; Catalyst: substituted dimethacrylate, 1,12-dodecane dimethacrylate, glass, silica, calcium hydroxide, calcium salt of 1-benzyl-5-phenyl-barbic-acid, sodium *p*-toluenesulfinate (574751)	43 vol %/12.5 μm
MCE	Maxcem Elite	Kerr Corp., Orange, CA, USA	GPDM, TEGDMA, fillers, activators, stabilizers, HEMA, cumene hydroperoxide, titanium dioxide, pigments (5427018)	69 wt %
BC	BisCem	Bisco Inc., Schaumberg, IL, USA	Di-HEMA phosphate, Tetra-EGDMA, glass (1500001067)	Base: 36 vol % (60 wt %); Catalyst: 40 vol % (62 wt %)/Base: 1.0 μm; Catalyst 3.5 μm
MLS	Multilink Speed	Ivoclar Vivadent, Schaan, Liechtenstein	Base: UDMA, TEGDMA; Catalyst: UDMA, TEGDMA, methacrylated phosphoric acid ester, PEGDMA, benzoyl peroxide (U18982)	Base: 75.0 wt %; Catalyst: 47.4 wt %
DL	Duo-Link	Bisco Inc., Schaumberg, IL, USA	Base: Bis-GMA, TEGDMA, UDMA, glass filler; Catalyst: Bis-GMA, TEGDMA, glass filler (1500003655)	38 vol % (60 wt %)/<1.0 μm

* Manufacturer supplied. Abbreviation of monomers in alphabetical order: Bis-GMA, bisphenol A diglycidyl methacrylate; Di-HEMA phosphate, di-2-hydroxyethyl methacryl hydrogenphosphate; GPDM, glycerol phosphate dimethacrylate; HEMA, 2-hydroxyethyl methacrylate; PEGDMA, polyethylene glycol dimethacrylate; TEGDMA, triethylene glycol dimethacrylate; Tetra-EGDMA, tetraethylene glycol dimethacrylate; UDMA, urethane dimethacrylate.

**Table 2:** Mean values (standard deviations) of surface energy parameters (mJ/m^2^) of the resin cements tested (*n* = 5).

Resin Cement	Curing Mode	*γ*_s_	*γ*_s_^LW^	*γ*_s_^+^	*γ*_s_^−^	*γ*_s_^AB^
RXU	Dual-cured	44.08 (0.33)^A^	43.58 (0.38)^A^	0.01 (0.002)^A^	11.20 (1.01)^A^	0.50 (0.09)^A^
	Self-cured	56.86 (0.84)^a^	49.01 (0.45)^a^	0.73 (0.06)^a^	21.35 (2.47)^ab^	7.85 (0.50)^a^
MCE	Dual-cured	37.90 (1.52)^B^	35.90 (0.97)^B^	0.05 (0.03)^B^	22.86 (0.94)^B^	1.99 (0.75)^BC^
	Self-cured	52.03 (0.74)^b^	39.46 (0.93)^b^	1.66 (0.35)^b^	23.96 (0.94)^a^	12.57 (1.49)^b^
BC	Dual-cured	42.98 (1.03)^A^	40.91 (1.08)^C^	0.04 (0.02)^B^	25.12 (2.30)^B^	2.08 (0.43)^BC^
	Self-cured	52.13 (2.06)^b^	33.75 (1.38)^c^	2.14 (0.61)^b^	39.98 (1.65)^c^	18.38 (2.97)^c^
MLS	Dual-cured	43.57 (0.61)^A^	41.78 (0.36)^C^	0.05 (0.01)^B^	15.87 (1.54)^C^	1.79 (0.27)^B^
	Self-cured	48.22 (1.14)^c^	43.84 (0.83)^d^	0.29 (0.02)^c^	16.75 (2.83)^b^	4.39 (0.37)^d^
DL	Dual-cured	46.27 (1.11)^C^	43.50 (1.14)^A^	0.17 (0.04)^C^	11.12 (1.01)^A^	2.77 (0.28)^C^
	Self-cured	46.24 (1.86)^c^	43.78 (0.74)^d^	0.16 (0.14)^c^	11.32 (0.84)^d^	2.46 (1.15)^e^

*γ*_s_, total surface energy; *γ*_s_^LW^, Lifshitz-van der Waals component; *γ*_s_^+^, acid component; *γ*_s_^−^, base component; *γ*_s_^AB^, acid/base component. Within a column, the same superscripted upper- and lower-case letters indicate statistically similar means within the dual- and self-cured specimens, respectively (*p* > 0.05). Within a column, underlining indicates statistically similar means between the two curing modes within each material (*p* > 0.05).
